# The differential role of magnetic resonance imaging in predicting surgical outcomes between children versus adults with temporal lobe epilepsy

**DOI:** 10.3389/fnins.2022.1037244

**Published:** 2022-11-16

**Authors:** Ke Xu, Xiongfei Wang, Jian Zhou, Yuguang Guan, Tianfu Li, Guoming Luan

**Affiliations:** ^1^Department of Neurosurgery, Sanbo Brain Hospital, Capital Medical University, Beijing, China; ^2^Department of Neurology, Sanbo Brain Hospital, Capital Medical University, Beijing, China; ^3^Department of Brain Institute, Center of Epilepsy, Beijing Institute for Brain Disorders, Beijing Key Laboratory of Epilepsy Research, Sanbo Brain Hospital, Capital Medical University, Beijing, China

**Keywords:** temporal lobe epilepsy, neurosurgery, neuropathology, magnetic resonance imaging, seizure outcome

## Abstract

**Objective:**

This study aims to investigate the clinical discrepancies and the different predictors of anterior temporal lobectomy (ATL) in children (<18 years at surgery) and adults (>18 years at surgery) with temporal lobe epilepsy (TLE).

**Materials and methods:**

A total of 262 patients (56 children and 206 adults) with TLE who underwent ATL were included in this study. The clinical variables, including patients’ characteristics, preoperative evaluations, pathology, surgical prognosis, and surgical predictors were assessed the discrepancies between TLE children versus adults using univariate and multivariate analyses. Kaplan-Meier survival analysis was used to calculate the probability of seizure freedom and AEDs withdrawal after ATL, and the difference between TLE children and adults was analyzed using the Log-Rank test.

**Results:**

There were significant differences including semiology, magnetic resonance imaging (MRI) examinations, numbers of preoperative AEDs, and pathologies between TLE children and adults (*P* < 0.05, *Q* < 0.05). The MRI-detected epileptic focus was the only independent predictor of seizure freedom (*P* = 0.002, *Q* = 0.036) in TLE children, and the concordance of MRI-detected focus with video-electroencephalography (video-EEG)-detected epileptic zone was the only variable associated with seizure freedom in TLE adults (*OR* = 2.686, 95% *CI* = 1.014–7.115, *P* = 0.047). The TLE children experienced a higher probability of AEDs withdrawal than adults after surgery (*P* = 0.005).

**Significance:**

There were remarkable differences in clinical manifestations, MRI examinations, number of preoperative AEDs, and pathologies between TLE children versus adults. TLE children had a higher possibility of AEDs withdrawal than adults after surgery. The favorable seizure outcome of ATL depended on the early complete resection of MRI-detected epileptogenic focus in TLE children, while the concordance of MRI-detected focus with EEG-detected epileptogenic zone was the only predictor of favorable seizure outcomes in TLE adults.

## Introduction

Temporal lobe epilepsy (TLE) is considered as the most common type of epilepsy that is refractory to antiepileptic drugs (AEDs) (Engel et al., 2003). Anterior temporal lobectomy (ATL) is a widely used surgical procedure with a seizure freedom rate from 62 to 83% (Mathon et al., 2015). Various studies have shown magnetic resonance imaging (MRI) – detected epileptogenic focus, particularly the presence of mesial temporal sclerosis, is a predictor of favorable outcomes in patients undergoing surgical treatment for TLE (Antel et al., 2002; McIntosh et al., 2004; Clusmann, 2008; Sun et al., 2015). However, significant discrepancies in presurgical, surgical, and postsurgical features between children and adults with TLE (Spencer and Huh, 2008; Ryvlin et al., 2014; Baud et al., 2018; Barba et al., 2021), and differences in the predictive role of MRI in TLE children versus adults remain poorly reported before (Barba et al., 2021). Furthermore, a large variation in the proportion of patients within the different pathological categories between children and adults has been reported before (Blumcke et al., 2017). Therefore, the predictors of seizure outcomes after ATL in children may not be appropriate for adults, due to these differences in clinical manifestations and pathology of TLE (Goldstein et al., 1996).

Few studies have described differences in clinical manifestations and predictors for ATL between TLE children and adults in single center (Lee et al., 2010; Baud et al., 2018; Cloppenborg et al., 2019; Barba et al., 2021). Accordingly, this study will answer the following questions: (1) What are the discrepancies in etiologies and clinical manifestations between TLE children and adults? (2) What are the differences in surgical effects on TLE children and adults with prognosis and AEDs withdrawal? (3) Does MRI have a different predictive role for surgical outcomes in children and adults with TLE? This study will highlight the differences in clinical manifestations, surgical prognosis, and surgical predictors between TLE children versus adults, and provide suitable surgical candidates for different TLE populations.

## Materials and methods

### Patients selection

Data of patients with TLE who had undergone surgery at Sanbo Brain Hospital, Capital Medical University from January 2009 to December 2019 were retrospectively recorded. Detailed data including demographic characteristics, clinical examinations, and post-surgical pathologies that can influence surgical outcomes were collected. This study was approved by the Ethics Committee of Sanbo Brain Hospital, Capital Medical University (SBNK-2017-15-01).

The exclusion criteria were as follows: (1) patients with drug-resistant extratemporal epilepsy; (2) patients who underwent extended ATL other than standard ATL (Spencer et al., 1984); (3) patients who underwent lesionectomy for the temporal lobe tumors; (4) patients who had a history of epilepsy surgery; (5) patients who had incomplete pathological tissue; (6) patients with the surgical pathology of encephalomalacia; (7) patients who had a follow-up for less than 24 months after surgery.

### Preoperative evaluation

The preoperative variables were collected from the medical records, which included sex, age at seizure onset, seizure duration, semiology, age at surgery, age at surgery, AEDs, history of febrile seizure (FS) (Menzler et al., 2011), MRI examinations, video-EEG, magnetoencephalography (MEG), [^18^F]-fluorodeoxyglucose positron emission tomography (^18^FDG-PET), stereoelectroencephalography (SEEG), and the side of surgery. Brain MRI of TLE patients was scanned with a 1.5 or 3.0-T scanner for T1, T2, and T2 fluid-attenuated inversion recovery (FLAIR) sequences. The standard 64-channel long-term video EEG monitoring was used in patients for at least 24 h. The video EEG was sampled at the rate of 1,024 samples and recorded in a double banana montage. The epileptogenic zone was defined according to the scalp or invasive EEG results, and the MRI results classified as normal, hippocampal sclerosis (HS), temporal lobe (TL) abnormalities (temporal blurring, dysplasia, or atrophy), both HS and TL abnormalities, and tumor. Accordingly, those patients with concordance or discordance of MRI and video-EEG results were distinguished, respectively. To accurately locate the epileptogenic zone, the MEG [102 patients (26 children; 76 adults)] can help to delineate the epileptogenic zone by localizing interictal epileptic spikes, PET [144 patients (26 children; 118 adults)] that can locate the hypometabolic regions, and SEEG [52 patients (6 children; 46 adults)] were also performed. After completion of the presurgical evaluation by neurosurgeons, neurologists, neuropsychologists, electrophysiologists, and neuroradiologists, the surgical decision was made.

### Surgical procedure

The purpose of ATL was to remove the epileptogenic zone and epileptogenic focus, and there is no difference in the surgical procedure between TLE children and adults. The standard ATL procedure included the resection of 3.0–3.5 cm from the anterolateral temporal lobe in the dominant hemisphere or the 4.0–4.5 cm of the temporal lobe in the non-dominant hemisphere. The resection of the mesial structure included the resection of the amygdala and the anterior 3.0 cm of the hippocampus. There was no difference in the resection of mesial structure between the dominant and non-dominant hemispheres (Spencer et al., 1984). For the patients with temporal lobe tumors, the ATL plus lesionectomy was performed.

### Surgical outcomes and complications

Patients were evaluated at 3 months postoperatively and yearly thereafter. The 16-h scalp-EEG and MRI were performed routinely. The surgical complications including intracranial hemorrhage, intracranial infection, and neurological dysfunction were recorded after surgery. The timing of the first postoperative seizure onset (beyond the first postoperative week for patients with acute postoperative seizures) was considered the time of seizure recurrence. Seizure outcomes were categorized according to the Engel classification system (Engel Jr, 1993). Favorable seizure outcomes were defined as Engel class I during the last 2 years of follow-up, and unfavorable seizure outcomes were defined as Engel class II–IV. For patients with seizure freedom of more than 2 years (Braun and Schmidt, 2014), the protocol for AEDs reduction was determined by the neurologist. The AEDs gradually tapered one by one. Thereafter, if patients had auras, seizures, or epileptiform abnormalities on scalp EEG results (Tang and Xiao, 2017), the AEDs were continued at the minimum doses without further tapering.

### Statistical analysis

Continuous variables were described using means ± standard deviations, and the categorical variables were described using frequencies and percentages. The difference between the adult and children’s subgroups was based on a cut-off age of 18 years at the surgery (Blumcke et al., 2017). Accordingly, the clinical variables, including patients’ characteristics, preoperative evaluations, pathology, and surgical prognosis were assessed the discrepancies between TLE children and adults using Pearson’s chi-square or Student *t-test*.

The cut-off variables were determined according to Youden’s index in a receiver operating characteristic curve analysis, and then variables were performed using Pearson’s chi-square or Fisher’s exact test to evaluate the predictors of a favorable outcome in TLE subgroups. The Benjamini-Hochberg false discovery rate (FDR) control to correct the final models for multiple comparisons. By default, this study used the first value in the list of variables as a reference category, after verifying that there were not too few cases in the chosen category. *P*-value and the FDR *Q*-value thresholds were set for significance. Finally, the variables showing a *Q* value < 0.05 in the univariate analysis were then into a multivariable logistic regression model in a backward manner for TLE adults and children, respectively. The odd ratios (ORs) and 95% confidence intervals (CIs) were calculated from the regression model.

Kaplan-Meier (KM) survival analysis was used to calculate the probability of seizure freedom and AEDs withdrawal after ATL, and the difference between TLE children and adults was analyzed using the Log-Rank test. The relationship between probable predictors and seizure freedom was also calculated by KM survival analysis in TLE adults and children, respectively. All analyses were performed using SPSS software (version 24.0, IBM, NY, USA), and a *P* value < 0.05 was considered statistically significant.

## Results

### Differences in patients’ characteristics between temporal lobe epilepsy subgroups

**FIGURE 1:**
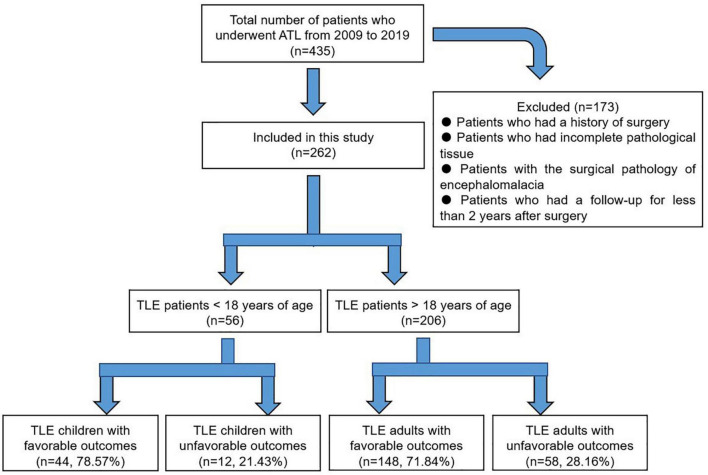
Outline of the study design and summary of outcomes. Favorable outcomes: patients with Engel class I during the last 2 years of follow-up; unfavorable outcomes: patients with Engel class II–IV during the last 2 years of follow-up.

Two hundred and sixty-two patients fulfilled the criteria and were analyzed in this study (56 children, 206 adults, Figure 1). The mean age at surgery was 11.39 ± 3.90 years in children and 28.16 ± 7.85 years in adults (*P* < 0.001; *Q* < 0.001); the mean age at seizure onset was 5.54 ± 4.37 years in children and 13.13 ± 9.78 years in adults (*P* < 0.001; *Q* < 0.001); the mean duration of epilepsy was 6.04 ± 3.77 years in children and 14.86 ± 9.39 years in adults (*P* < 0.001; *Q* < 0.001). In addition, the detailed comparison of semiology (Fisher et al., 2017), types of AEDs, and history of FS between TLE children and adults were described in Table 1.

**TABLE 1:** The difference in demographic characteristics between temporal lobe epilepsy (TLE) children and adults.

Demographic characteristics	Children	Adults	*P*-value	*Q*-value
Sex (male)	33 (58.93%)	108 (52.43%)	0.451	0.501
Age at seizure onset	5.54 ± 4.37	13.13 ± 9.78	0.000*	0.000*
Duration of epilepsy	6.04 ± 3.77	14.86 ± 9.39	0.000*	0.000*
Auras	16 (28.57%)	110 (53.39%)	0.001*	0.003*
Impaired awareness	43 (76.79%)	142 (68.93%)	0.321	0.458
Automatisms	33 (58.93%)	145 (70.39%)	0.109	0.182
Secondary-GTCS	23 (41.07%)	129 (62.62%)	0.006*	0.017*
FS	7 (12.50%)	43 (20.87%)	0.183	0.282
**Numbers of preop AEDs**				
Two types	39 (69.64%)	189 (91.75%)	0.001*	0.003*
Three types	17 (30.36%)	17 (8.25%)		
**MRI results**				
Normal	2 (3.57%)	7 (3.39%)	0.000*	0.000*
HS	13 (23.21%)	130 (63.11%)		
Additional TL abnormalities	13 (23.21%)	37 (17.96%)		
Both HS and additional TL abnormalities	3 (5.36%)	16 (7.77%)		
Tumor	25 (44.64%)	16 (7.77%)		
IEDs on unilateral temporal lobe	31 (55.36%)	127 (61.65%)	0.442	0.521
Ictal onset on unilateral temporal lobe	17 (30.36%)	80 (38.83%)	0.47	0.495
MEG on unilateral temporal lobe	9 (*N* = 26, 34.62%)	44 (*N* = 76, 57.89%)	0.045*	0.1
PET on unilateral temporal lobe	19 (*N* = 26, 73.08%)	61 (*N* = 118, 51.69%)	0.047*	0.095
SEEG implantation	6 (10.71%)	46 (22.33%)	0.085	0.155
Age at surgery	11.39 ± 3.90	28.16 ± 7.85	0.000*	0.000*
Surgery on the left side	29 (51.79%)	109 (52.91%)	0.881	0.881
**Neuropathology**				
FCD type I	12 (21.42%)	48 (23.30%)	0.000*	0.000*
HS	4 (7.14%)	106 (51.46%)		
FCD type IIIa	25 (44.64%)	32 (15.53%)		
Tumors	15 (26.78%)	16 (7.77%)		
Others	0 (0.00%)	4 (1.94%)		
Surgical complications	7 (12.50%)	18 (8.70%)	0.395	0.504
Favorable outcomes	44 (78.57%)	148 (71.84%)	0.395	0.493

HS, hippocampal sclerosis; GTCS, generalized tonic–clonic seizure; FS, febrile seizure; ATL, anterior temporal lobectomy; MRI, magnetic resonance imaging; TL, temporal lobe; IEDs, interictal epileptic discharges; AEDs, antiepileptic drugs; MEG, magnetoencephalography; PET, positron emission tomography; SEEG, stereo-electroencephalography; FCD, focal cortical dysplasia.

*P < 0.05. Bold: TLE children experienced earlier age at seizure onset, shorter duration of epilepsy, fewer auras, and fewer secondary-GTCS than those in TLE adults. The TLE children took more AEDs than those in TLE adults. More MRI-detected HS was observed in TLE adults, while more MRI-detected tumors were observed in TLE children. The TLE adults experienced elder age at surgery than those in TLE children. More neuropathology of HS was observed in TLE adults, while more neuropathology of tumors was observed in TLE children.

### Differences in preoperative evaluation between temporal lobe epilepsy subgroups

Magnetic resonance imaging examinations were obtained in 56 children (2 were normal, 13 were HS, 13 were TL abnormalities, 3 were both HS and TL abnormalities, and 25 were temporal tumors) and 206 adults (7 were normal, 130 were HS, 37 were TL abnormalities, 16 were both HS and TL abnormalities, and 16 were temporal tumors; *P* < 0.001, *Q* < 0.001). During the video-EEG monitoring, interictal epileptic discharges (IEDs) were recorded in all patients with 31 (55.4%) children and 127 (61.7%) adults arising at unilateral temporal lobe (*P* = 0.442). The ictal onset rhythms (IORs) were detected in unilateral temporal lobe in 17 (30.4%) children and 80 (38.9%) adults (*P* = 0.470). The MEG spikes sources locating at unilateral temporal lobe were observed in 9 (34.6%) children and 44 (57.9%) adults (*P* = 0.045; *Q* = 0.100). The hypometabolic regions of PET locating in the unilateral temporal lobe were found in 19 (73.1%) children and 61 (51.7%) adults (*P* = 0.047; *Q* = 0.095). SEEG implantation was performed in 6 (10.7%) children and 46 (22.3%) adults (*P* = 0.085, Table 1).

### Differences in surgical prognosis between temporal lobe epilepsy subgroups

The surgery on the left side was performed in 29 (51.8%) children and 109 (52.9%) adults (*P* = 0.881). There were no operative or perioperative deaths. Surgical complications occurred in 7 (12.5%) children; 1 (1.8%) had intracranial hemorrhage, 3 (5.4%) had intracranial infection, 1 (1.8%) had transient hemiplegia, and 2 (3.6%) had transient aphasia. Surgical complications were observed in 18 (8.7%) adults; 2 (0.9%) had intracranial heamorrhage, 1 (0.5%) had a subarachnoid hemorrhage, 2 (0.9%) had an intracranial infection, 4 (1.9%) had transient hemiplegia, and 9 (4.4%) had transient aphasia. Quadrantanopia was not considered a surgical complication in this study. There was no significant difference of surgical complications between TLE children and adults (*P* = 0.395, Table 1).

After a follow-up period of 2–5 years (mean 3.47 ± 1.91 years), 44 (78.6%) children achieved seizure freedom, and 148 (71.8%) adults achieved seizure freedom after ATL (*P* = 0.395, Table 1). For these seizure freedom patients, AEDs had completely discontinued in 32 (72.7%) children and 73 (35.4%) adults. Two (6.3%) children and 8 (10.9%) adults experienced seizure recurrence after AEDs withdrawal.

### Differences in neuropathology between temporal lobe epilepsy subgroups

Surgical specimens were processed for histological analysis. The HS was diagnosed in 4 (7.1%) children and 106 (51.5%) adults. Focal cortical dysplasia (FCD) type I was diagnosed in 12 (21.4%) children (6 FCD type Ia, 6 FCD type Ib) and 48 (23.3%) adults (21 FCD type Ia, 27 FCD type Ib). Fifteen (26.8%) children were diagnosed with temporal tumors (14 gangliogliomas WHO grade I, 1 dysembryoplastic neuroepithelial tumor WHO grade I), and 16 (7.8%) adults were diagnosed with tumors (9 gangliogliomas WHO grade I, 5 dysembryoplastic neuroepithelial tumors WHO grade I, and 2 astrocytomas WHO grade II). Besides, 25 children (44.6%) and 32 adults (15.5%) were diagnosed with FCD type IIIa (*P* < 0.001; *Q* < 0.001, Table 1).

### Prognostic factors of seizure outcomes

**FIGURE 2:**
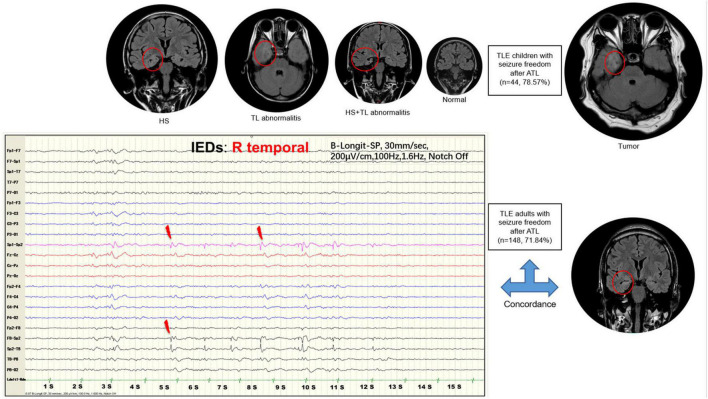
The differential role of magnetic resonance imaging (MRI) in predicting surgical outcomes between temporal lobe epilepsy (TLE) children versus adults. The favorable seizure outcome of anterior temporal lobectomy (ATL) depended on the early complete resection of MRI-detected epileptogenic focus in TLE children. The size of the image represented the contribution to the favorable outcomes after surgery. The concordance of MRI with interictal epileptic discharges (IEDs) results was the only predictor of favorable seizure outcomes in TLE adults. The scalp EEG were sampled at the rate of 1,024 samples, and the typically bandpass filtered for viewing between 1.6 and 100 Hz. The data was recorded in a double banana montage, and the sensitivity was 200 μV/cm.

In the univariate analysis of postoperative seizure outcomes, the duration of epilepsy (≤7.5 years), secondary-GTCS, MRI examinations, and neuropathology showed a significant difference in TLE children (*P* < 0.05, Table 2). However, the MRI examinations were the only independent predictor of favorable seizure outcomes after the FDR correction (*Q* = 0.036, Table 2). The TLE children with MRI-detected tumor had a better prognosis after ATL, while those with MRI-detected TL abnormalities were suggested to experience a worse seizure outcome (Figure 2).

**TABLE 2:** Children’s demographic characteristics and their relationship with seizure outcomes.

Demographic characteristics	Favorable outcome (*N* = 44)	Unfavorable Outcome (*N* = 12)	*P*-value	*Q*-value
	**Number (%)**	**Number (%)**		
Sex (male)	25 (56.82%)	8 (66.67%)	0.539	1.033
Age at seizure onset (≤7.5 years)	27 (61.36%)	9 (75.00%)	0.382	0.879
Duration of epilepsy (≤7.5 years)	33 (75.00%)	5 (41.67%)	0.028*	0.215
Age at ATL (≤14.5 years)	40 (90.91%)	9 (75.00%)	0.14	0.403
Monthly seizure frequency (ł10 times)	27 (61.36%)	7 (58.33)	0.849	0.849
Automatisms	11 (25.00%)	5 (41.67%)	0.257	0.657
Impaired awareness	36 (81.82%)	7 (58.33%)	0.088	0.337
Autism	29 (65.91%)	4 (33.33%)	0.054	0.248
Secondary-GTCS	15 (34.09%)	8 (66.67%)	0.042*	0.241
FS	6 (13.64%)	1 (8.33%)	0.622	0.953
**Numbers of preop AEDs**				
Two types	31 (70.45%)	8 (66.67%)	0.801	0.921
Three types	13 (29.54%)	4 (33.33%)		
**MRI results**				
Normal	2 (4.54%)	0 (0.00%)	0.002*	0.046*
HS	9 (20.45%)	4 (33.33%)		
Additional TL abnormalities	6 (13.63%)	7 (58.33%)		
Both HS and additional TL abnormalities	3 (6.81%)	0 (0.00%)		
Tumor	24 (54.54%)	1 (8.33%)		
IEDs on unilateral temporal lobe	25 (56.82%)	6 (50.00%)	0.674	0.912
Ictal onset on unilateral temporal lobe	14 (31.82%)	3 (25.00%)	0.649	0.933
Concordance of MRI with IEDs	19 (43.18%)	4 (33.33%)	0.539	1.033
Concordance of MRI with ictal onset	11 (25.00%)	2 (16.67%)	0.544	0.962
MEG on unilateral temporal lobe	6 (*N* = 18, 33.33%)	3 (*N* = 8, 37.50%)	0.837	0.875
PET on unilateral temporal lobe	15 (*N* = 20, 75.00%)	4 (*N* = 6, 66.67%)	0.686	0.877
SEEG implantation	3 (6.82%)	3 (25.00%)	0.105	0.345
Surgery on the left side	22 (50.00%)	7 (58.33%)	0.609	1.005
**Neuropathology**				
FCD type I	7 (15.91%)	5 (41.67%)	0.022*	0.253
HS	2 (4.54%)	2 (16.67%)		
FCD type IIIa	11 (25%)	4 (33.33%)		
Tumors	24 (54.54%)	1 (8.33%)		

HS, hippocampal sclerosis; GTCS, generalized tonic–clonic seizure; FS, febrile seizure; ATL, anterior temporal lobectomy; MRI, magnetic resonance imaging; TL, temporal lobe; IEDs, interictal epileptic discharges; AEDs, antiepileptic drugs; MEG, magnetoencephalography; PET, positron emission tomography; SEEG, stereo-electroencephalography; FCD, focal cortical dysplasia.

*P < 0.05. Bold: The TLE children with MRI-detected tumors had a better prognosis after surgery than those with other MRI results.

After the FDR correction of univariate analysis in the TLE adults, the following factors were associated with favorable seizure outcomes: IEDs on the unilateral temporal lobe and concordance of MRI with IEDs (*Q* < 0.05, Table 3). Therefore, these two variables were recruited into the logistic regression model. The regression analysis revealed that the concordance of MRI with IEDs results was the only predictor of favorable seizure outcomes in TLE adults (OR = 0.2.686, 95% CI = 1.014–7.115, *P* = 0.047, Table 4).

**TABLE 3:** Adults’ demographic characteristics and their relationship with seizure outcomes.

Demographic characteristics	Favorable outcome (*N* = 148)	Unfavorable outcome (*N* = 58)	*P*-value	*Q*-value
	**Number (%)**	**Number (%)**		
Sex (male)	75 (50.68%)	33 (56.89%)	0.421	0.509
Age at seizure onset (≤11.5 years)	72 (48.65%)	24 (41.38%)	0.347	0.532
Duration of epilepsy (≤25 years)	130 (87.84%)	47 (81.03%)	0.207	0.476
Age at ATL (≤22.5 years)	35 (23.65%)	12 (20.69%)	0.649	0.711
Monthly seizure frequency (≥10 times)	58 (39.19%)	26 (44.82%)	0.459	0.528
Auras	83 (56.08%)	27 (46.55%)	0.218	0.455
Secondary-GTCS	105 (70.96%)	37 (63.79%)	0.318	0.522
Automatisms	108 (72.97%)	37 (63.79%)	0.235	0.45
Secondary-GTCS	90 (60.81%)	39 (67.24%)	0.391	0.529
FS	35 (23.65%)	8 (13.79%)	0.117	0.336
**Numbers of preop AEDs**				
Two types	136 (91.89%)	53 (91.38%)	0.904	0.945
Three types	12 (8.11%)	5 (8.62%)		
**MRI results**				
Normal	4 (2.70%)	3 (5.17%)	0.058	0.222
HS	102 (68.92%)	28 (48.28%)		
Additional TL abnormalities	23 (15.54%)	14 (24.14%)		
Both HS and additional TL abnormalities	8 (5.41%)	8 (13.79%)		
Tumor	11 (7.43%)	5 (8.62%)		
IEDs on unilateral temporal lobe	101 (68.24%)	26 (44.83%)	0.002*	0.023*
Ictal onset rhythms on unilateral temporal lobe	64 (43.24%)	16 (27.59%)	0.038*	0.291
Concordance of MRI with IEDs	87 (58.78%)	18 (31.03%)	0.001*	0.023*
Concordance of MRI with ictal onset	55 (37.16%)	11 (18.97%)	0.042*	0.242
MEG on unilateral temporal lobe	29 (*N* = 47, 61.70%)	15 (*N* = 29, 51.72%)	0.392	0.501
PET on unilateral temporal lobe	48 (*N* = 87, 55.17%)	13 (*N* = 31, 41.94%)	0.205	0.524
SEEG implantation	33 (22.29%)	13 (22.41%)	0.986	0.986
Surgery on the left side	75 (50.68%)	34 (58.62%)	0.304	0.537
**Neuropathology**				
FCD type I	32 (21.62%)	16 (27.59%)	0.387	0.556
HS	76 (51.35%)	30 (51.72%)		
FCD type IIIa	27 (18.24%)	5 (8.62%)		
Tumors	11 (7.43%)	5 (8.62%)		
Others	2 (1.35%)	2 (3.45%)		

HS, hippocampal sclerosis; GTCS, generalized tonic–clonic seizure; FS, febrile seizure; ATL, anterior temporal lobectomy; MRI, magnetic resonance imaging; TL, temporal lobe; IEDs, interictal epileptic discharges; AEDs, antiepileptic drugs; MEG, magnetoencephalography; PET, positron emission tomography; SEEG, stereo-electroencephalography; FCD, focal cortical dysplasia.

*P < 0.05. Bold: The TLE adults with IEDs on the unilateral temporal lobe or concordance of MRI with IEDs experienced better surgical outcomes.

**TABLE 4:** Predictors of seizure outcome in adults on multivariate analysis.

Variables	OR	95% CI	*P*-value
IEDs on unilateral temporal lobe	1.241	0.481–3.2	0.655
Concordance of MRI with IEDs results	2.686	1.014–7.115	0.047*

IEDs, interictal epileptic discharges; MRI, magnetic resonance imaging; OR, odds ratio; CI, confidence interval.

*P < 0.05. Bold: The regression analysis revealed that the concordance of MRI with IED results was the only predictor of favorable seizure outcomes in TLE adults.

### Kaplan-Meier analysis of seizure-free survival

**FIGURE 3:**
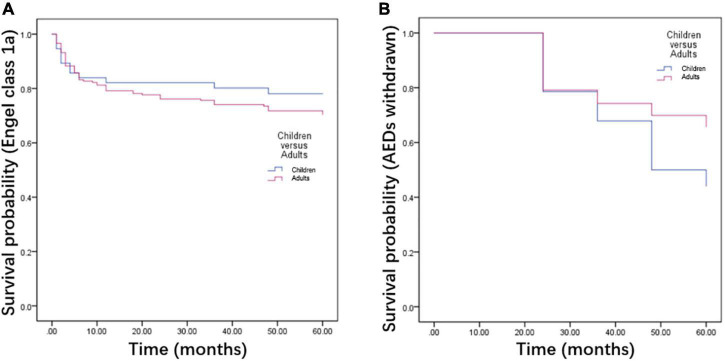
Kaplan-Meier survival curves of children and adults with temporal lobe epilepsy (TLE). There was no significant difference in seizure recurrence between TLE children and adults **(A)**. The TLE adults experienced a lower probability of antiepileptic drugs (AEDs) withdrawal than TLE children after surgery **(B)**.

**FIGURE 4:**
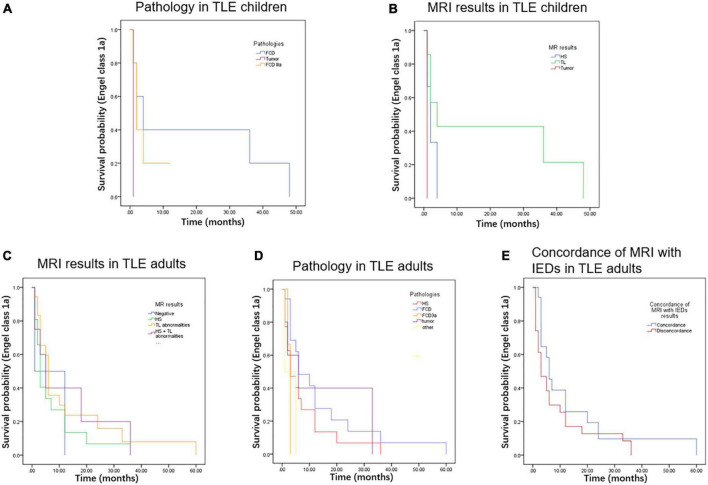
The Kaplan-Meier estimates of the probability of cumulative seizure-free survival in months were not a significant difference in those TLE children with different pathologies **(A)**, different MRI results **(B)**, in those TLE adults with different MRI results **(C)**, different pathologies **(D)**, and concordance of MRI with IEDs **(E)**.

The KM survival curves were made to evaluate the seizure-free survival and AEDs withdrawal between children and adults. The results revealed that there was no significant difference in seizure-free survival between children and adults (*P* = 0.353, Figure 3A). However, TLE adults experienced a lower probability of AEDs withdrawal than children (*P* = 0.005, Figure 3B). The KM estimates of the probability of cumulative seizure-free survival in months were no significant difference in those with or without prognostic factors in TLE subgroups (*P* > 0.05, Figure 4).

## Discussion

This study analyzed the significant differences in clinical manifestations, pathologies, and prognoses of TLE children versus adults, which promoted the understanding of the discrepancies between TLE children and adults. In addition, the predictive factor of MRI which affect the short and long-term prognosis after ATL was important for the appropriate selection and counselling in different TLE populations.

### Differences in clinical manifestations and pathologies between temporal lobe epilepsy children and adults

As the results showed in this study, the TLE adults experienced more presence of auras, automatisms, and secondary-GTCS than those TLE children. In addition, the types of lesions in MR and pathology were significant differences in the TLE subgroups. This study revealed that HS was the most common pathology in the brain tissue of epileptic adults, and the FCD was the most common pathology among children, which was consistent with a previous study (Blumcke et al., 2017). The distinct pathology and long-term seizure duration promote the more complex epileptic network in TLE adults, which could induce comprehensive semiology. Therefore, TLE children and adults should be studied separately to reduce the bias in the prognostic analysis (Barba et al., 2021). A previous study found very similar clinical manifestations and responses to surgical treatment in TLE children and adults (Asadi-Pooya and Sperling, 2015). Conversely, the other multicentre analysis demonstrated significant differences in several presurgical, surgical, and postsurgical features between adults and children (Barba et al., 2021). These discrepancies might be explained by differences in inclusion criteria.

### Predictive role of magnetic resonance imaging in the temporal lobe epilepsy children

Anterior temporal lobectomy was the choice to provide better access to the tumor or if the epileptogenic area was much larger than the tumor itself in children (Cataltepe et al., 2005). The completeness of tumor resection determined seizure outcomes in children (Khajavi et al., 1999; Lopez-Gonzalez et al., 2012), and several investigators had reported that seizure freedom was more than 80% of patients after the completeness resection of the tumor (Boon et al., 1991; Britton et al., 1994; Cataltepe et al., 2005). The relationship between tumors and epileptogenic focus was still unclear (Zhang et al., 2020). Furthermore, it was found that HS in 56% of pediatric patients with temporal lobe tumors (Drake et al., 1987), and it would be speculated that the hippocampus was often epileptogenic because of abnormal synaptic reorganization of the hippocampus induced by seizures secondary to temporal tumors (Cataltepe et al., 2005). In this study, 96% of children with temporal tumor-related epilepsy achieved a seizure-free outcome after ATL, which was proposed that ATL could completely remove the suspicious HS and potential FCD surrounding tumors in TLE children. Conversely, the children with TL abnormalities on MRI experienced worse seizure outcomes than those with tumors on MRI. Therefore, the early complete resection was prompted to improve surgical outcomes for children with temporal tumor-related epilepsy. In contrast, the TLE children with TL abnormalities on MRI should be considerable for ATL. Other predictive factors in TLE children including age at seizure onset, duration of epilepsy, and the presence of secondary-GTCS (Cohen-Gadol et al., 2006; Elliott et al., 2013; He et al., 2020; Barba et al., 2021) were not statistically significant after FDR correction in this study. The unified surgery and statistical methods would influence the results.

### Predictive role of concordance of magnetic resonance with interictal epileptic discharges in temporal lobe epilepsy adult

The relationship between the predictive role of video-EEG and MRI on seizure outcome was still debated. Several studies detected a significant predictive contribution of video-EEG, particularly in patients with HS or with negative MRI examinations (Holmes et al., 2000; Schulz et al., 2000; Sun et al., 2015). In contrast, other studies demonstrated that the discrepancies between video-EEG and MRI examinations indicated a poor surgical outcome (Vinton et al., 2007; Bote et al., 2008). In the present study, we highlighted the concordance of MRI-detected focus with IEDs-detected epileptic zone, which played a predictive role in the favorable seizure outcome. However, this predictive role was neither in MRI nor video-EEG findings alone in TLE adults, and this concordance was also insignificant for the surgical outcome in TLE children. Therefore, there was a more extensive epileptogenic zone in TLE adults than in children. TLE children should undergo the early complete resection of epileptogenic focus, while TLE adults should undergo the resection of an area where the epileptogenic focus was concordant with the epileptogenic zone.

### Differences in surgical prognosis between temporal lobe epilepsy children and adults

The results of this study revealed that the percentage of achieving seizure freedom was higher in children than in adults after ATL (78.57 vs. 71.84%), but the possibility of seizure recurrence in patients was not significantly different between children and adults at both 2-year and last follow-up in Log-rank test. Besides, there was only a slight drop in seizure freedom in TLE children overtimes (from 78.57 to 73.21%). Several studies revealed that the percentage of children with seizure freedom remained unchanged at the last follow-up (Miserocchi et al., 2013; Ormond et al., 2019). Besides, the probability of AEDs withdrawal in TLE children (72.72%) was higher than that in TLE adults (35.44%), which was consistent with the previous study (Barba et al., 2021). Previous studies supported a minimum seizure freedom period of 2 years before considering AEDs withdrawal (Beghi et al., 2013; Braun and Schmidt, 2014). Moreover, apart from age > 30 years and longer disease duration, other factors associated with a higher risk of seizure recurrence after AEDs withdrawal were persistent auras, seizure relapse before withdrawal, and postoperative EEG abnormalities (Shih and Ochoa, 2009). Therefore, this study indicated the short duration of epilepsy, complete resection of the epileptic lesion, and non-epileptiform discharge on postoperative EEG in TLE children could achieve a higher possibility of AEDs withdrawal and a lower rate of seizure recurrence after AEDs than that in TLE adults.

### Limitations

There were some limitations to this study. First, the consequences of this study were limited by its retrospective nature and relatively short follow-up after surgery of TLE patients. Second, the sample size of TLE children was relatively small which could influence the results. Finally, the neuropsychological analysis was unavailable in the study because of incomplete postoperative information.

## Conclusion

There were remarkable differences in clinical manifestations, MRI examinations, and pathologies between TLE children versus adults. TLE children had a higher possibility of AEDs withdrawal than adults after surgery. The favorable seizure outcome of ATL depended on the early complete resection of MRI-detected epileptogenic focus in TLE children, while the concordance of MRI-detected focus with IEDs-detected epileptogenic zone was the predictor of favorable seizure outcomes in TLE adults.

## Data availability statement

The original contributions presented in this study are included in the article/supplementary material, further inquiries can be directed to the corresponding author.

## Ethics statement

The studies involving human participants were reviewed and approved by Sanbo Brain Hospital, Capital Medical University (SBNK-2017-15-01). Written informed consent from the participants’ legal guardian/next of kin was not required to participate in this study in accordance with the national legislation and the institutional requirements.

## Author contributions

KX: formal analysis, investigation, and writing the original draft. XW: validation. YG: methodology. JZ: investigation. TL: validation. GL: conceptualization, supervision, and writing – review and editing. All authors contributed to the article and approved the submitted version.
